# Correction: *Candida albicans PPG1*, a serine/threonine phosphatase, plays a vital role in central carbon metabolisms under filament-inducing conditions: a multi-omics approach

**DOI:** 10.1371/journal.pone.0308954

**Published:** 2024-08-12

**Authors:** Mohammad Tahseen A. L. Bataineh, Nelson Cruz Soares, Mohammad Harb Semreen, Stefano Cacciatore, Nihar Ranjan Dash, Mohamad Hamad, Muath Khairi Mousa, Jasmin Shafarin Abdul Salam, Mutaz F. Al Gharaibeh, Luiz F. Zerbini, Mawieh Hamad

The raw data underlying Figs 1 and 2 are missing from the Supporting Information. The authors have provided the data as GSE263609, accessible here: https://www.ncbi.nlm.nih.gov/geo/query/acc.cgi?acc=GSE263609.

The Data Availability statement is therefore incorrect. The correct statement is: All relevant data are within the manuscript and its Supporting Information file or are available at GEO with accession number GSE263609.

With this correction, all relevant data are now provided.
